# Few-Shot Fine-Grained Forest Fire Smoke Recognition Based on Metric Learning

**DOI:** 10.3390/s22218383

**Published:** 2022-11-01

**Authors:** Bingjian Sun, Pengle Cheng, Ying Huang

**Affiliations:** 1 School of Technology, Beijing Forestry University, Beijing 100083, China; 2 Department of Civil, Construction, and Environmental Engineering, North Dakota State University, Fargo, ND 58102, USA

**Keywords:** forest fire smoke, fine-grained recognition, metric learning, few-shot learning

## Abstract

To date, most existing forest fire smoke detection methods rely on coarse-grained identification, which only distinguishes between smoke and non-smoke. Thus, non-fire smoke and fire smoke are treated the same in these methods, resulting in false alarms within the smoke classes. The fine-grained identification of smoke which can identify differences between non-fire and fire smoke is of great significance for accurate forest fire monitoring; however, it requires a large database. In this paper, for the first time, we combine fine-grained smoke recognition with the few-shot technique using metric learning to identify fire smoke with the limited available database. The experimental comparison and analysis show that the new method developed has good performance in the structure of the feature extraction network and the training method, with an accuracy of 93.75% for fire smoke identification.

## 1. Introduction

Forest fires often cause significant economic losses and ecological damage, which are classified as highly destructive disasters. In recent decades, as global warming increases and the climate changes, forest fires have become very frequent in certain regions such as Canada and the western U.S. Once a forest fire spreads, it burns a large number of trees, and in many circumstances, it is difficult for the forest to return to its original appearance. Forest fires also may induce soil erosion and environmental pollution, lead to deaths or habitat loss for wildlife, threaten the biodiversity of the planet’s ecology [1], and bring immeasurable loss of property and life to people and society [2].

Thus, to prevent the spread of forest fires and mitigate their impacts, it is of extreme importance to detect fires at an early stage. Currently, the forest fire detection methods of a forest fire can be divided into three categories: smoke detection [3], flame detection [4], or a combination of both [5,6,7]. However, due to the large amount of vegetation in the forest environment, when a forest fire occurs, the flames are easily blocked in the early stages of the fire, limiting the application of early forest fire detection using the flame detection method.

**Figure 1:**
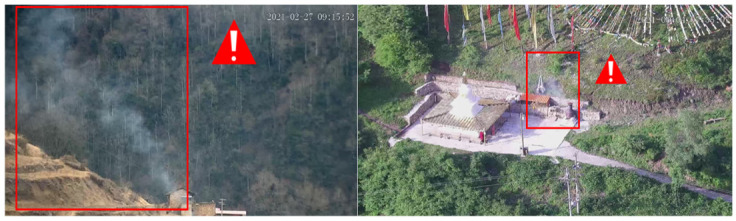
Non-hazardous smoke in a forest monitoring scenario causing an alarm when using existing smoke detection methods.

One the other hand, the smoke produced spreads over time and is not affected by vegetation. Therefore, it can be a good candidate for the detection and warning for the early stages of forest fire. However, in addition to the smoke produced by fires, non-hazardous smoke is produced by other activities such as home cooking, factory manufacturing, etc., as shown in Figure 1. If non-hazardous smoke in the monitoring regions is detected and triggers a forest fire alarm, it is a false alarm known as an In-Smoke False Positive problem.

Currently, to detect smokes in a forest, there are two available technologies: smoke sensors [8] and visual cameras [9]. The smoke sensors detect the physicochemical properties of smoke, which is challenging for applications in open forest monitoring scenarios and cannot distinguish between the different smoke classes produced by forest fire or a non-hazardous causes. Visual cameras record images [10] or videos [11,12,13,14,15], and the smoke detection is performed by feature extraction to identify, locate, or segment smoke in the recorded images or videos.

The traditional visual detection method directly uses artificially designed features. For example, the commonly used feature descriptions in the mainstream traditional image-based smoke detection methods [16,17] include color, texture, edge and other features. These cannot be used to distinguish different types of smoke, so the traditional image-based smoke detection method cannot avoid the false positive issues when different classes of smoke are present in the detection scenes. Compared with image-based smoke detection, the traditional video-based smoke detection can contain dynamic features. However, there is no significant difference between the dynamic features of various classes of smokes. Thus, the traditional video-based smoke detection can only be used to distinguish smoke and non-smoke, and cannot avoid the false positives in the smoke class.

Recently, based on the recorded images/videos from visual detection tools, researchers tend to use deep learning methods to detect smoke through learning features from the training data followed by detecting smoke using these extracted features. For visual smoke detection based on deep learning methods, depending on the nature of the detection tasks, the accuracy varies with the composition of the data set, the design of the network structure, and the learned features. Commonly, the different types of detection tasks are as follows: dichotomy tasks with smoke and non-smoke [18,19]; three-category tasks with smoke, non-smoke, and flame [20]; four-category tasks with smoke, smoke with fog, non-smoke, and non-smoke with fog [21]; five-category tasks with smoke, clouds, fog, trees, and cliffs [22]; target detection tasks for locating smoke [23,24,25]; and image segmentation tasks that demarcate the smoke area [26,27]. It can be seen that the common tasks do not divide smokes into different classes and the network cannot extract the characteristics that distinguish different classes of smokes. Thus, the current deep learning-based smoke detection methods cannot avoid false positives in the smoke classes.

**Figure 2:**
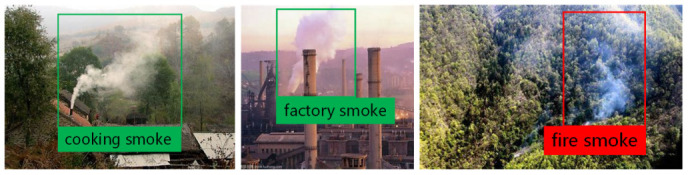
Fine-grained smoke identification screens out non-hazardous smoke.

However, non-hazardous smoke is very common in forest monitoring scenarios. The frequent false alarms bring concerns to the relevant personnel with unnecessary panic and waste of human resources, reduce the trust in the fire detection system, and may result in the situation that the responsible firefighters ignore the fire alarms and miss actual forest fires. Targeting to reduce the false alarm rate of forest fire smokes, for the first time, this paper proposes a few-shot fine-grained smoke identification method using metric learning to screen out the non-hazardous forest fire smokes, as shown in Figure 2. To address the constraint that limited smoke data can be obtained for different smoke classes, this paper first establishes a few-shot dataset with a small number of images from multiple smoke classes including cooking, factory, and forest fire smokes. Then, suitable models are designed to identify the three smoke classes using a feature extraction network and loss function with its corresponding training method. Specifically, for the feature extraction network, this paper compares a variety of classical networks, and analyzes the relationship between the performance of the network and the ratio of its parameter quantities to the number of layers. For the loss function, this paper compares the contrast loss functions, including contrastive loss, triplet loss, circle loss and instance loss, that are commonly used in metric learning [28] and the cross-entropy loss function that is commonly used in probability learning. Two support strategies for using training samples and using new samples are compared when contrast loss is used.

The rest of this paper is arranged as follows: the methods of this article are introduced in detail in Section 2; Section 3 presents the experimental results and an analysis of the methods proposed in this article, and finally, the findings are summarized in Section 4.

## 2. Methodology

### 2.1. Task Definition

In this paper, considering that forest fire smoke detection scenes are mostly disturbed by cooking smoke generated by residences and factory smoke, the fine-grained classification includes three categories: cooking smoke, factory smoke and fire smoke. The feasibility of this task is based on considerations of human experience. Different types of smoke can be distinguished by the judgment of smoke sources, which is similar to fine-grained recognition tasks in other fields. Therefore, the metric learning applicable to other fields is also applicable to the smoke field. However, unlike fine-grained recognition tasks such as face recognition, bird species judgment, and automobile brand judgment, the types of smoke are more limited and deterministic. Thus, networks are usually not tested by new kinds, and the few-shot situation in smoke fine-grained identification tasks is not generated by new species, but rather due to the low probability nature of fire smoke itself. Therefore, in this task, a small sample fire smoke types is also used in the training process of the network, which is different from the conventional few-shot learning process based on metric learning, and is an adaptive change made after fully considering the characteristics of the task.

### 2.2. Dataset

**Figure 3:**
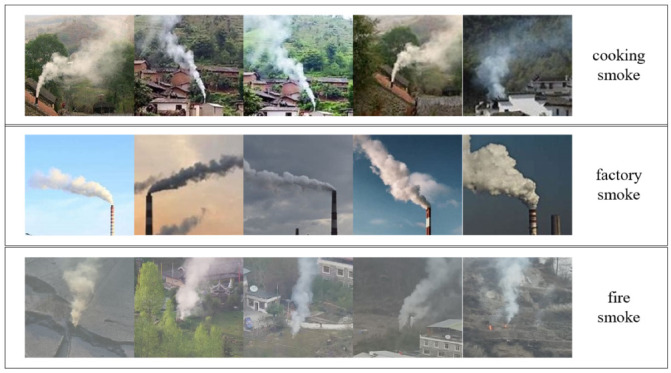
Partial data from the dataset used by the fine-grained smoke identification task.

The dataset used in this paper is a self-built dataset, part of which is derived from network pictures, and the other part of the data is derived from real forest fire smoke detection scenarios. Considering the identification task needs, the dataset includes three types of smoke images, namely, cooking smoke, factory smoke and fire smoke. The cooking smoke and factory smoke are categorized as non-hazardous smokes and do not cause fire alarms. As in the case of sparse real smoke data, the dataset was constructed with only a small number of samples, in which the training dataset had 9 images for each category making a total of 27; in the test set there are 16 pictures in each category making a total of 48 pictures. Figure 3 shows the example data from the dataset. It is desirable to obtain a fine-grained smoke recognition model from a small number of samples.

### 2.3. Network Structure

**Figure 4:**
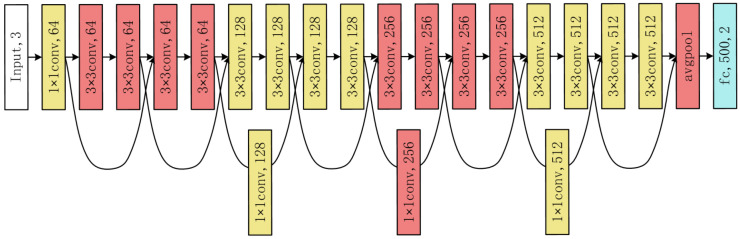
Network structure diagram of ResNet18.

Due to the small number of samples, it is very important to select the appropriate feature extraction networks for training. An optimal network structure requires a suitable network depth for the proposed task, which ranges from 6 to 30, and an appropriate network size within that depth range. The ResNet18 [29] network structure, as shown in Figure 4, is selected in this paper as it falls within the depth range and is the best performing moderately sized network structure. The network consists of an 18-layer network structure with a short circuit connection between each residual module to prevent gradients from disappearing or exploding.

### 2.4. The Loss Function and Its Corresponding Training Process

Deep metric learning has achieved promising results in face recognition tasks. The feature extraction network can determine whether two faces are the same person through learning local key features, such as a person’s facial features, that distinguish a person’s face. This is similar to the smoke fine-grained recognition task, but the key feature that distinguishes smoke is the source of smoke, which is learned by training with smoke samples.

**Figure 5:**
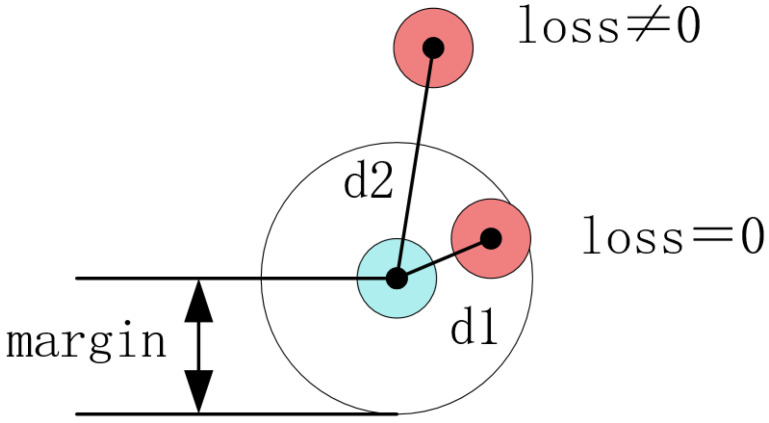
The function of the parameter m.

In metric learning, the results of classification are based on the Euclidean distance between the eigenvectors. If the eigenvectors distance of two samples is less than the threshold, they are considered to be of the same category; otherwise, they are considered to belong to different categories. Thus, the goal of metric learning is to make the eigenvectors of the same sample closer and the eigenvectors of different classes of samples farther apart. To achieve this, it is necessary to determine the loss function in training. In deep metric learning, contrast loss has a concise form and is easy to converge during training, so this paper uses contrast loss to train the network. For contrast loss, L_contrastive_, the training sample consists of two training images. As shown in Equation (1), when the categories of the two training images are the same, the true label of the training data is 0; otherwise, the true label of the training data is 1. The two eigenvectors are obtained for the two images in the sample through the feature extraction network:

(1) $$L_{\mathrm{Contrastive}}=\left(1-Y\right)\frac{1}{2}{\left(D_{W}\right)}^{2}+\left(Y\right)\frac{1}{2}{\left\{\max\left(0,m-D_{W}\right)\right\}}^{2}$$

where Y is the true label of the sample, D_W_ is the Euclidean distance between two eigenvectors in the sample, and m is a hyper-parameter, as shown in Figure 5, which controls the minimum distance between samples of different classes. The loss value is greater than zero when the distance between samples of the same class is greater than zero, and when the distance between different classes is less than the margin, loss is incurred.

**Figure 6:**
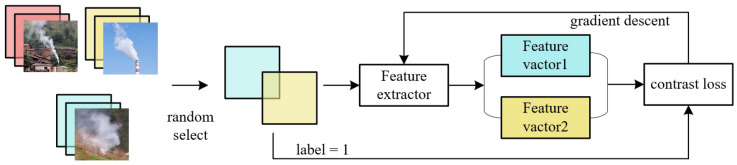
Network training process.

Figure 6 shows the training process of the network, where a pair of images I_a_ (i = i_a_, j = j_a_) and I_b_ (i = i_b_, j = j_b_) are randomly selected in the training set T_ij_ to constitute a training sample S, where j_a_ ≠ j_b_. When i_a_ = i_b_, the two images belong to different categories, and the training sample S is true labeled 0; otherwise, it is 1. Next, I_a_ and I_b_ are entered into the feature extraction network D, and the corresponding feature vectors F_a_ = D(I_a_) and F_b_ = D(I_b_) are obtained. Then, the Euclidean distance between F_a_ and F_b_ is calculated, and the Dw and corresponding L_contrastive_ are obtained. The parameter m of the network is obtained by optimizing the argmin(m) L_contrastive_.

### 2.5. Model Framework for Fine-Grained Smoke Identification Tasks

**Figure 7:**
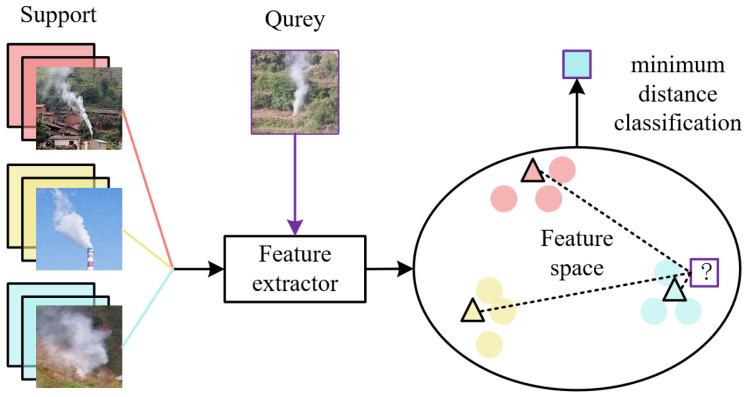
Model framework for smoke fine-grained identification task.

After determining the network structure and training, the model framework of the task is shown in Figure 7. The metric model proposed in this paper uses the ResNet network as feature extractor D, and uses the feature center C_i_ provided by the training set to guide the classification during the testing phase. In this paper, the lighter ResNet18 in the ResNet family was adopted to minimize overfitting of the depth model in the case of small sample sizes.

Feature Extractor D learns how to extract features F of smoke to distinguish between different categories of smoke. For example, its extracted features may be related to the source of smoke: cooking smoke is often above a roof, factory smoke rise from high chimneys, and forest fire smoke usually rises in grassy groves. The trained feature extractor results in the feature vectors being distributed in the feature space according to the category; that is, the sample feature vectors of different classes are far away, and the sample feature vectors of the same class are close. Feature center C_i_ is an important basis for classification, and after each test sample obtains a feature vector, it is necessary to calculate the distance from feature center C_i_ to determine its category.

Specifically, the feature center C_i_ is generated by the training set T_ij_, and i = 1,2,3 is the category number, and j = 1, …, N_i_ is the sample number. The eigenvector of the training set is first obtained by D, which is F_ij_ = D(T_ij_), and C_i_is the average of F_ij_ in the j dimension. I is the input test image, and its feature vector F is generated by D: F = D(I). The distance d_i_ of F to C_i_ is then estimated and finally the category of I C(I) = argmin (i)(d_i_) is determined.

## 3. Case Studies and Discussion

To validate the proposed new method in this paper, case studies on the experimental database described in Section 2.2 were performed, analyzed and compared using the three main elements in the method.

### 3.1. Results

**Figure 8:**
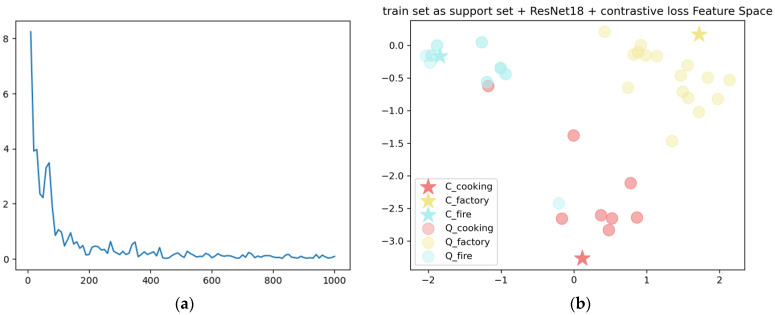
(**a**) Network training curve, and (**b**) distribution of feature vectors of test samples in feature space.

**Figure 9:**
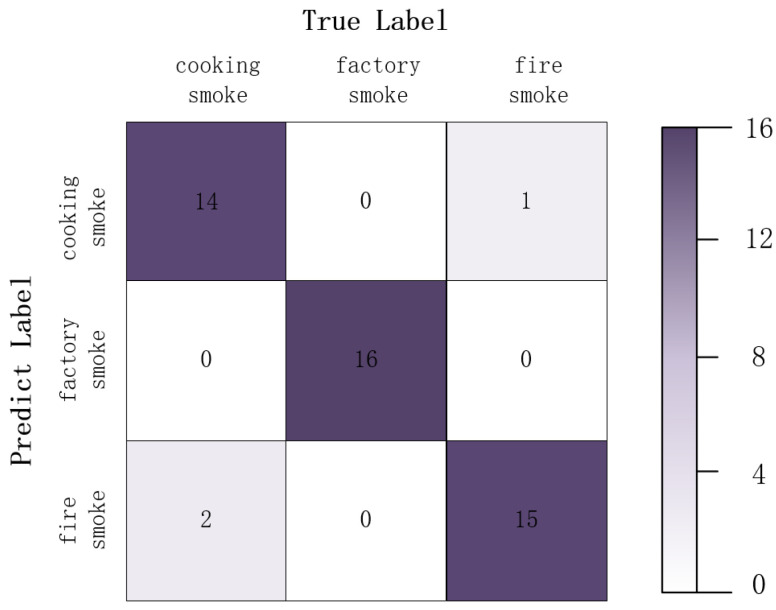
Confusion matrix.

Figure 8a shows the training curve obtained by training the network ResNet18 using the training dataset and the contrast loss. The training set is taken as the support set, and the support set and the test set are extracted by feature extraction network to obtain the feature vector. Its distribution in the feature space is shown in Figure 8b, where the star data points represent the feature center C_i_ and the circular data points represent the feature vectors of the test data. It can be seen that the distribution of the feature vectors of each category has a clear tendency of intra-class aggregation and inter-class separation. Figure 9 shows the results of the test in the form of a confusion matrix. It can be seen from the matrix that the recognition effect of factory smoke is better, which may be because the locations where factory smoke appears tend to have a simpler background, and the characteristics of the smoke source site are more obvious. There is still a certain error rate in the network for the distinction between cooking smoke and fire smoke. It may be caused by areas of overlap between human dwellings and vegetation in a forest environment, which results in a similar background for the two types of smoke. In addition, cooking smoke has similar characteristics to those of fire smoke. It is not highly significant in terms of color, texture, etc.

**Figure 10:**
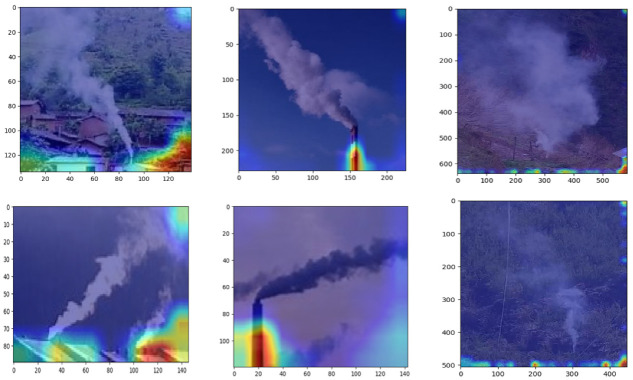
Attentional salient feature map of some test samples.

To further examine the reliability of the network, we visualized the salient feature map of the network, and Figure 10 shows the salient feature map of example data in the network. As can be seen in Figure 10, the network is largely based on the source of the smoke for type judgment; for example, for cooking smoke, the network is based on the house, for factory smoke, the network is based on the chimney, and for fire smoke, the network is based on grass.

### 3.2. Network Optimization and Discussions

**Figure 11:**
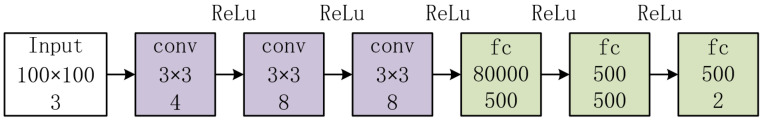
Structure of a CNN network used to ensure data coherence.

The primary and important element in the model architecture is the backbone network, and in this paper, two issues need to be considered to determine the backbone network to be used. First of all, the number of samples is very small, requiring the network to have fewer parameters. Otherwise, the network would overfit. Secondly, for the fine-grained smoke recognition task, it is necessary to extract the semantic features of the smoke source from the image, which has certain requirements for the depth of the network. When the number of network layers is small, the semantic features may not be extracted. Under these constraints, a network is expected to have better performance when it is deeper and the number of parameters is small. However, when the network is too sparse, it also faces the issue of semantic feature extraction difficulties. Therefore, to optimize the network, we first limited the number of layers of the network to between 6 and 30 according to the nature of the task, and then selected a variety of networks in this range for testing, as shown in Table 1, including GoogleNet, MobileNet, ResNet18, Shallow CNN, AlexNet, and VGGNet. Among these networks, to ensure the coherence of the data, a shallow CNN network is established based on the ResNet18 network and the AlexNet network. Figure 11 shows the specific structure of the shallow CNN network, where the input data has a size of 100 × 100 and the number of channels is three. The convolutional layer has a convolutional kernel size of 3 × 3 and an output channel count of four. The fully connected layer has an input length of 80,000 and an output length of 500. These networks have different ratios of parameter quantities to the number of layers, with NL representing the number of layers of the network and NP representing the parameter quantity of the network.

**Figure 12:**
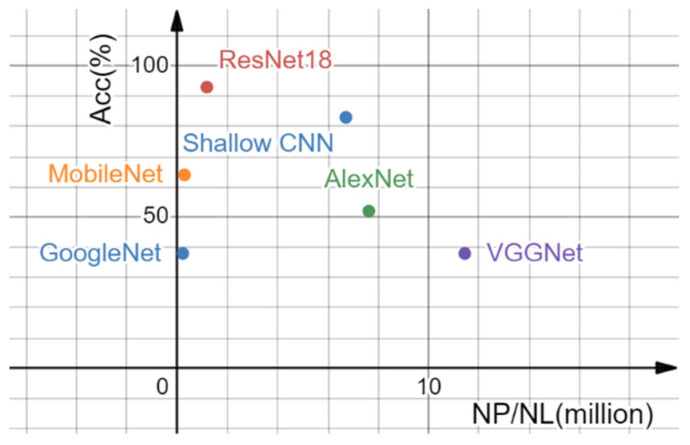
Relationship between NP/NL and accuracy of the network.

Figure 12 shows the relationship between the NP/NL ratio and the accuracy of the tested networks. It can be seen that when NP/NL is too large or too small, the network accuracy declines. ResNet18 performed best in terms of detection accuracy, validating the selection of ResNet18 as a network in this paper.

In addition to the backbone model, the loss function is another important element in the model architecture. To verify the effectiveness of metric learning in fine-grained identification tasks, this paper compares the loss functions including contrastive loss, triplet loss, circle loss and instance loss that are commonly used in metric learning and the cross-entropy loss function that is commonly used in probability learning. Among them, it is found through experiments that the design of triplet loss, circle loss and instance loss increases the convergence difficulty of the network, especially in the case of few training samples, which results in a low test accuracy of the network. In the case of triplet loss, circle loss and instance loss, the test accuracy of the network is 64.2%, 54.8% and 64.2%, respectively. Therefore, hereafter we mainly discuss contrastive loss and cross-entropy loss. When using the cross-entropy loss function, the output dimension of the last fully connected layer of the network is the number of categories, which is three in this task. The vector is normalized using the softmax activation function, on the basis of which the principle of probability maximum is used for classification. In metric learning, to determine the category of test samples, support samples are necessary. In the usual metric learning task, due to the limitations of the task itself, the type of test sample is a new type for the network, so the support samples are also new samples. However, in the fine-grained smoke recognition task, there is no need to keep the fire smoke as a new type; therefore, in order to give full play to the learning ability of the network, we directly use the training samples as support samples. For example, the embedding vectors of all fire smoke training samples are averaged to obtain the support vector of the fire smoke type as the support vector of factory smoke and cooking smoke.

**Figure 13:**
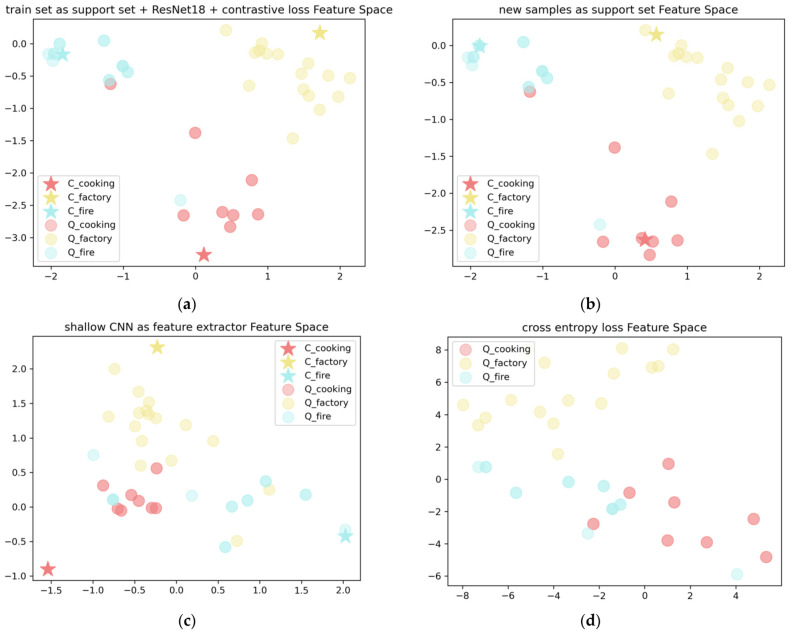
The comparison results are presented in the form of feature spatial distribution map.

For a test sample, after obtaining its embedded vector, its type is determined by calculating the distance from each type support vector. Table 2 and Figure 13 show the results of the comparison. The sub-plots shown in Figure 13 correspond to the items in Table 2, which are the distributions of the category-centered feature vectors and the test data feature vectors in the feature space. It can be seen by comparing Figure 13a,b that the longer spacing is more conducive to the differentiation of categories. This shows that the use of the network-learned training set as a support set is conducive to the network obtaining a more accurate category-centered feature vector. From Figure 13c, it can be seen that due to the use of shallow CNN as feature extraction networks, the feature vectors of the test data cannot be well clustered with the centers of each class, indicating that the shallow CNN learning limited the selection of features. In Figure 13d, although the test data show good convergence, since the loss function does not have an interval constraint and does not have a support set, the samples near the boundaries of each category are prone to misclassification.

## 4. Conclusions and Future Work

In this paper, fine-grained smoke recognition under the few-shot condition was achieved using the metric learning method. Specifically, ResNet18 was selected as the feature extractor and the contrast loss was applied for training. The feature centers of the training set were introduced during the testing phase to guide classification. The developed method obtained a detection accuracy of 93.75% on the test set of the self-built data set. By applying the method of this paper to the coarse-grained smoke detection method, the non-fire smoke in the detection results can be further screened out, thereby reducing the risk of false positives within the smoke class in the forest fire monitoring scenario.

In the future, to improve the overall accuracy of forest fire smoke detection tasks, it is necessary to further improve the interclass accuracy of smoke detection. Additionally, the attention of network still needs to be optimized for various types of smoke, especially fire smoke.

## Figures and Tables

**Table 1:** Model parameters and their accuracy.

Network	NL	NP (million)	NP/NL (million)	Accuracy (%)
GoogleNet [30]	22	5	0.227	38
MobileNet [31]	17	5	0.294	64
ResNet18	18	21	1.183	**93**
Shallow CNN	6	40	6.709	83
AlexNet [32]	8	60	7.62	52
VGGNet [33]	16	180	11.43	38

**Table 2:** Results of substitution comparison of major elements in the model.

Model (Support Strategy, Feature Extractor and Loss Function)	Accuracy
training set as support set + ResNet18 + contrastive loss	0.9375
training set as support set → new samples as support set	0.9167
ResNet18 as feature extractor → shallow CNN as feature extractor	0.8333
contrastive loss → cross-entropy loss	0.8125

## Data Availability

Not applicable.

## References

[1] Fu X. (2021). The impact of forest fire on forest ecosystems. Mod. Agric. Res..

[2] Chen Y.J., Lei Y.J. (2022). Hazards of forest fires and fire prevention measures. Mod. Agric..

[3] Khan S., Muhammad K., Mumtaz S., Baik S.W., De Albuquerque V.H.C. (2019). Energy-efficient deep CNN for smoke detection in foggy IoT environment. IEEE Internet Things J..

[4] Majid S., Alenezi F., Masood S., Ahmad M., Gündüz E.S., Polat K. (2022). Attention based CNN model for fire detection and localization in real-world images. Expert Syst. Appl..

[5] Saponara S., Elhanashi A., Gagliardi A. (2021). Real-time video fire/smoke detection based on CNN in antifire surveillance systems. J. Real-Time Image Process..

[6] Valikhujaev Y., Abdusalomov A., Cho Y.I. (2020). Automatic fire and smoke detection method for surveillance systems based on dilated cnns. Atmosphere.

[7] Jadon A., Omama M., Varshney A., Ansari M.S., Sharma R. (2019). FireNet: A specialized lightweight fire & smoke detection model for real-time IoT applications. arXiv.

[8] Li H.D. (2021). Design of wireless fire early warning system based on composite sensor. Master’s Thesis.

[9] Gu K., Xia Z., Qiao J., Lin W. (2019). Deep dual-channel neural network for image-based smoke detection. IEEE Trans. Multimed..

[10] Muhammad K., Khan S., Palade V., Mehmood I., De Albuquerque V.H.C. (2019). Edge intelligence-assisted smoke detection in foggy surveillance environments. IEEE Trans. Ind. Inform..

[11] Xu G., Zhang Y., Zhang Q., Lin G., Wang Z., Jia Y., Wang J. (2019). Video smoke detection based on deep saliency network. Fire Saf. J..

[12] Lin G., Zhang Y., Xu G., Zhang Q. (2019). Smoke detection on video sequences using 3D convolutional neural networks. Fire Technol..

[13] Gao Y., Cheng P.L. (2019). Forest fire smoke detection based on visual smoke root and diffusion model. Fire Technol..

[14] Gao Y., Cheng P.L. (2021). Full-Scale Video-Based Detection of Smoke from Forest Fires Combining ViBe and MSER Algorithms. Fire Technol..

[15] Lou L.M., Chen F., Cheng P.L., Huang Y. (2022). Smoke root detection from video sequences based on multi-feature fusion. J. For. Res..

[16] Feng X.H., Cheng P.L., Chen F. (2022). Full-Scale Fire Smoke Root Detection Based on Connected Particles. Sensors.

[17] Liu B.W., Sun B.J., Cheng P.L., Huang Y. (2022). An embedded portable lightweight platform for real-time early smoke detection. Sensors.

[18] Tao C., Zhang J., Wang P. Smoke detection based on deep convolutional neural networks. Proceedings of the 2016 International Conference on Industrial Informatics-Computing Technology, Intelligent Technology, Industrial Information Integration (ICIICII).

[19] Sun X., Sun L., Huang Y. (2021). Forest fire smoke recognition based on convolutional neural network. J. For. Res..

[20] Sheng D., Deng J., Xiang J. (2021). Automatic smoke detection based on SLIC-DBSCAN enhanced convolutional neural network. IEEE Access.

[21] He L., Gong X., Zhang S., Wang L., Li F. (2021). Efficient attention based deep fusion CNN for smoke detection in fog environment. Neurocomputing.

[22] Li T., Zhu H., Hu C., Zhang J. (2022). An attention-based prototypical network for forest fire smoke few-shot detection. J. For. Res..

[23] Li J., Zhou G., Chen A., Wang Y., Jiang J., Hu Y., Lu C. (2022). Adaptive linear feature-reuse network for rapid forest fire smoke detection model. Ecol. Inform..

[24] Zheng X., Chen F., Lou L., Cheng P., Huang Y. (2022). Real-Time Detection of Full-Scale Forest Fire Smoke Based on Deep Convolution Neural Network. Remote Sens..

[25] Hu Y., Zhan J., Zhou G., Chen A., Cai W., Guo K., Li L. (2022). Fast forest fire smoke detection using MVMNet. Knowl.-Based Syst..

[26] Yuan C., Liu Z., Zhang Y. (2019). Learning-Based Smoke Detection for Unmanned Aerial Vehicles Applied to Forest Fire Surveillance. J. Intell. Robot. Syst..

[27] Khan S., Muhammad K., Hussain T., Del Ser J., Cuzzolin F., Bhattacharyya S., De Albuquerque V.H.C. (2021). Deepsmoke: Deep learning model for smoke detection and segmentation in outdoor environments. Expert Syst. Appl..

[28] Hoffer E., Ailon N. (2015). Deep metric learning using triplet network. International Workshop on Similarity-Based Pattern Recognition.

[29] He K., Zhang X., Ren S., Sun J. Deep residual learning for image recognition. Proceedings of the IEEE Conference on Computer Vision and Pattern Recognition.

[30] Szegedy C., Liu W., Jia Y., Sermanet P., Reed S., Anguelov D., Erhan D., Vanhoucke V., Rabinovich A. Going deeper with convolutions. Proceedings of the IEEE Conference on Computer Vision and Pattern Recognition.

[31] Howard A.G., Zhu M., Chen B., Kalenichenko D., Wang W., Weyand T., Andreetto M., Adam H. (2017). Mobilenets: Efficient convolutional neural networks for mobile vision applications. arXiv.

[32] Krizhevsky A., Sutskever I., Hinton G.E. (2017). Imagenet classification with deep convolutional neural networks. Commun. ACM.

[33] Simonyan K., Zisserman A. (2014). Very deep convolutional networks for large-scale image recognition. arXiv.

